# Nitric oxide mediates ET-1-induced-inhibition of NPPB-sensitive Cl^−^ currents in the early distal convoluted tubule of the mouse kidney

**DOI:** 10.1016/j.jbc.2026.111202

**Published:** 2026-01-23

**Authors:** Lixia Hu, Hao Zhang, Ao Xiao, Haiwen Qiu, Xinxin Meng, Yi You, Mingxiao Wang

**Affiliations:** Department of Physiology, Zhuhai Campus of Zunyi Medical University, Zhuhai, Guangdong, China

**Keywords:** endothelin-1, early distal convoluted tubule, ClC-K2, nitric oxide, sodium-chloride cotransporter

## Abstract

Endothelin-1 (ET-1) from renal-tubule-epithelial-cells inhibits NaCl reabsorption *via* ET_B_ receptor in an autocrine manner, and inhibition of ET_B_ receptors leads to salt-sensitive-hypertension. In the distal convoluted tubule (DCT), NaCl enters the cell *via* NaCl-cotransporter (NCC) and Cl^−^ exits the cell in part by ClC-K2 channels, which play a role in regulating With-No-lysine kinase 4 (WNK4). The study aims to explore whether ET-1-induced inhibition of NaCl absorption is also achieved by inhibiting the basolateral Cl^−^ channels in the DCT. Patch-clamp and immunoblotting assessed ET-1 effects on DCT Cl^−^ channels and NCC. Immunofluorescence images detected ETB-receptor expression in parvalbumin-positive DCT. Application of ET-1 decreased NPPB-sensitive Cl^−^ currents and reduced 10-pS Cl^−^ channel activity (ClC-K2), defined by NPo (A product of channel number and open probability); this effect was absent in the presence of ET_B_ receptor inhibitor. Application of L-NAME (nitric oxide synthase inhibitor), ODQ (soluble guanosine cyclase inhibitor) or Bay-60-7550 (phosphodiesterase-2-inhibitor) *perse* did not affect Cl^−^ channels, but it abolished the inhibitory effect of ET-1. In contrast, application of NO-donor or cGMP inhibited ClC-K2 channel activity of the DCT. Moreover, ET-1 had no additional inhibitory effect of ET-1 on ClC-K2 in the presence of NO-donor or cGMP. Immunoblotting demonstrated that ET-1 treatment (200 nM) of renal cortex decreased NCC phosphorylation and total NCC expression, an effect that was abolished by inhibiting phosphodiesterase-2 but not by KT-5823 (PKG-inhibitor). In conclusion, ET-1 inhibits NCC and ClC-K2 in DCT by NO-sGMP-phosphodiesterase-2-dependent pathway.

The distal convoluted tubule (DCT) is responsible for reabsorbing 5%-10% of the filtered NaCl load (1). In DCT1, Na^+^ and Cl^−^ from the tubule lumen are transported into cells through sodium chloride cotransporter (NCC). Na^+^ is then transported into the interstitial fluid by the Na^+^-K^+^-ATPase, while Cl^−^ is transferred through ClC-K2 Cl^−^ channels in the basolateral membrane (2). Patch-clamp experiments conformed that ClC-K2 is a 10-pS and demonstrates sensitivity to 5-nitro-2-(3-phenylpropylamino)-benzoic acid (NPPB) (1, 2, 3). It has been reported that a decrease in ClC-K2 currents in the DCT leads to an increase of [Cl^-^]_i_, thereby inhibiting NCC activity *via* the with no lysine kinase-4 (WNK4) pathway (4). The importance of ClC-K2 in regulating NCC has been demonstrated that NCC activity is almost completely inhibited in *Clcnk2*^*−/−*^ mice (5). The endothelin (ET) family includes ET-1, ET-2, and ET-3, with ET-1 playing a significant role in regulating functions (6). In the kidney, the renal tubules are able to synthesize ET-1 *via* autocrine mechanisms to promote urinary sodium excretion (7, 8, 9). The diuretic and natriuretic effects of ET-1 are mediated through the activation of ET_B_ receptors, which stimulate nitric oxide (NO) release. Previous study demonstrated that blocking ET_B_-receptors in the medullary collecting duct (CD) leads to urinary sodium retention and salt-sensitive hypertension (10), possibly by stimulating epithelial-Na^+^-channel (ENaC). However, the mechanisms other than inhibiting ENaC in the collecting duct may also be responsible for urinary sodium retention in the animal models without ET_B_ (11, 12). In this regard, because DCT plays an important role in regulating NaCl absorption and ET_B_ receptor may be expressed in the early DCT (DCT1) (13), it raises the question whether ET-1 regulates NaCl reabsorption in the DCT1 *via* inhibiting NCC and ClC-K2. Thus, the aim of the present study is to examine the effect of ET-1 on NPPB-sensitive Cl^−^ currents and NCC and the mechanism by nitric oxide which ET-1 inhibits NCC and ClC-K2.

## Results

### ET_B_ is expressed in DCT1 and regulates ClC-K2

**Figure 1:**
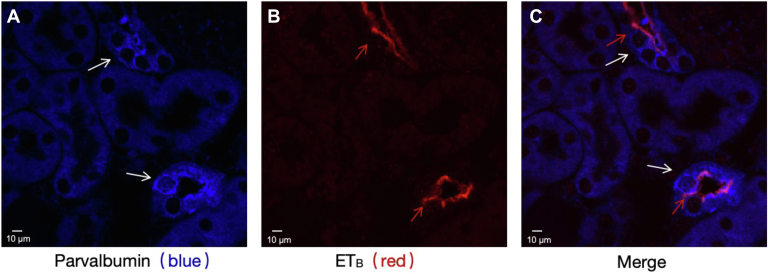
**ET_B_ receptor is expressed in the membrane of the DCT1**. *A*, parvalbumin (PV) immunostaining (*blue*) in the mouse kidney with 20× magnification. *B*, ET_B_ receptors immunostaining (*red*) in the mouse kidney with 20× magnification. *C*, fluorescence microscope image showing the merged staining of PV (*blue*) and ET_B_ (*red*) receptors. The DCT1 are indicated by *white arrows*. ET_B_ receptors where denoted by a *red arrow*, are present not only on the apical membrane but also on the lateral membrane of the DCT1. PV, parcalbumin. Scale bar, 10 μm.

**Figure 2:**
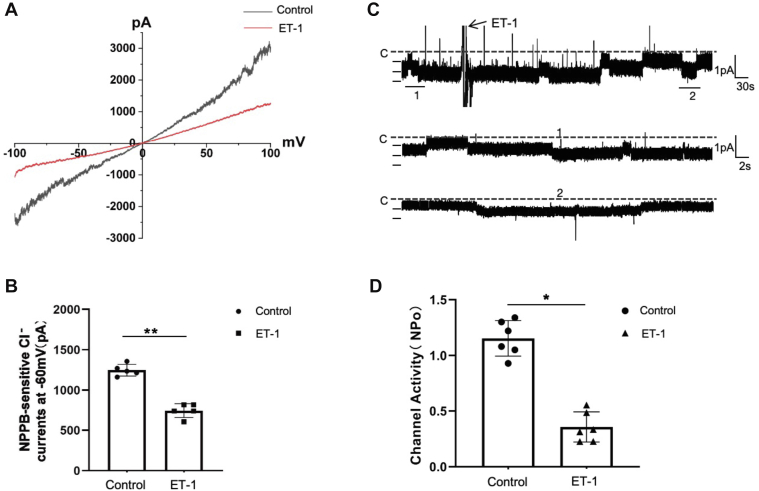
**ET-1 inhibits the NPPB-sensitive Cl^−^ currents in DCT1**. *A*, whole-cell recording showing the NPPB-sensitive Cl^−^ currents in different DCT1 cells before (*black line*) and after ET-1 (*red line*) with ramp protocol. In ET-1 group, the DCT1 was teated with 200 nM ET-1 for 5-7 min before examine the NPPB-sensitive Cl^−^ currents. *B*, a graph displays each NPPB-sensitive Cl^−^ currents with whole-cell recording at −60 mV. *C*, Single-channel recording demonstrating the effect of 200 nM ET-1 on the 10 pS Cl channels in the DCT1. The experiments were conducted in a cell-attached patch with a holding potential of −60 mV. The top trace illustrates the experimental timeline, with two segments of the trace, marked by numbers, expanded to exhibit the fast temporal resolution. The channel closed state is denoted by a *dotted line* and the letter 'C'. *D*, the bar graph summarizes the effect of ET-1 on the 10 pS Cl^−^ channel. All values are means ± SD. The significance is evaluated using a paired Student's *t* test. An *asterisk* indicates a significant difference compared with the control value (∗: *p* < 0.05,∗∗: *p* < 0.01).

Although ET_B_ receptors were not detected in the DCT of rats (14), both ET_A_ and ET_B_ receptors mRNA have been identified in the mouse DCT (13). Now employed immunofluorescence microscope to examine whether the ET_B_ receptors were expressed in DCT1. As shown in Figure 1, ET_B_ receptors is expressed in parvalbumin (PV)-positive tubules, an indication of early distal convoluted tubule (DCT1) (15, 16). After demonstrating ET_B_ is expressed in DCT1. We next investigated the NPPB-sensitive Cl^−^ currents in isolated DCT1 by whole-cell recording. The recording method for NPPB-sensitive Cl^−^ currents is detailed in the Fig. S2. Figure 2*A* presents a trace of NPPB-sensitive Cl^−^ currents measured with the ramp protocol from −100 to 100 mV. The NPPB-sensitive Cl^−^ currents are −1248.22 ± 72.22 pA at −60 mV (black line, n = 5). Then, we examined the impact of ET-1 on NPPB-sensitive Cl^−^ currents in diverse DCT1 cells. We pre-added ET-1 (200 nM) to the bath for 5-7 min and then measured the NPPB-sensitive Cl^−^ currents. As shown in Figure 2*A*, following administration of ET-1, the currents decreased to −745.62 ± 87.52 pA at −60 mV (red line, n = 5). We also employed the single-channel patch-clamp recording technique to study the effects of ET-1 on ClC-K2. Figure 2*C* is a representative recording showing that ET-1 (200 nM) inhibits the 10-pS Cl^−^ channel in a cell-attached patch, and with no difference in sexual (Fig. S4). The channel activity, defined by NPo, ranged from 1.14 ± 0.25 to 0.32 ± 0.16 (n = 5). The calculation process for determining Cl^−^ channel NPo is detailed in the Fig. S3. Figure 2, *B* and *D* presents the results from individual data, showing the effect of ET-1 on the Cl^−^ channel. These experiments suggest that ET-1 inhibits ClC-K2 channel currents in DCT1.

### ET_B_ receptors mediate the inhibitory effect of ET-1 on NPPB-sensitive Cl^−^ currents

**Figure 3:**
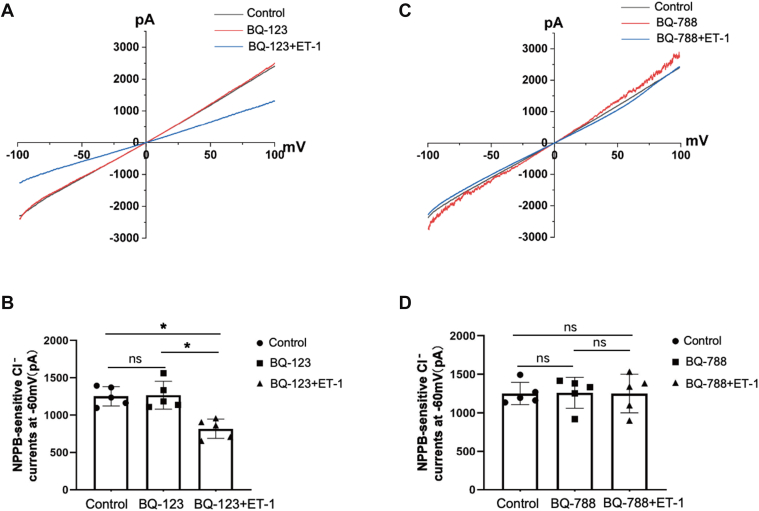
**Effect of ET-1 on NPPB-sensitive Cl^−^currents in the presence of ET-1 receptors**. *A*, a whole-cell recording examines the effect of 200 nM ET-1 on NPPB-sensitive Cl^−^ currents in DCT1 in the presence of 1 μM BQ-123. *B*, a bar graph summarizes the findings recorded at −60 mV. *C*, a whole-cell recording examines the effect of ET-1 on NPPB-sensitive Cl^−^ currents in DCT1 in the presence of 1 μM BQ-788. *D*, a bar graph illustrates each experiment of NPPB-sensitive Cl^−^ current examined at −60 mV. In the currents trace, the black line represents the normal NPPB-sensitive Cl^−^ currents without any treatment. The red line indicates the effect of BQ-123 or BQ-788 on NPPB-sensitive Cl^−^ currents, with the inhibitors added to the bath beforehand. The *blue line* represents the currents with pre-added inhibitors and ET-1. Each experiment was conducted on different DCT1 cells. All values are means ± SD. The significance is evaluated using a one-way ANOVA method. "ns" means no significance. ∗ indicates *p* < 0.05. BQ-123, ET_A_ receptors inhibitor; BQ-788, ET_B_ receptors inhibitor.

Previous research have established that the diuretic and natriuretic effects of ET-1 are primarily mediated through ET_B_ receptors (8, 9). To investigate the function of ET-1 receptors in regulating the inhibitory effect of ET-1, we examined the effect of ET-1 on NPPB-sensitive Cl^−^ currents in the DCT1, treated with the ET_A_ receptor antagonist (BQ-123) or ET_B_ receptor antagonist (BQ-788). Figure 3 illustrates NPPB-sensitive Cl^−^ currents using the ramp protocol with different treatments. The experiments were conducted on different DCT1 cells. The inhibitors and ET-1 were pre-added to the bath for 5 to 7 min. It is shown that neither BQ-123 (1 μM) nor BQ-788 (1 μM) affects the NPPB-sensitive Cl^−^ currents, the Cl^−^ currents are −1265.14 ± 186.65 pA (BQ-123, Fig. 3*A*, red line) and −1260.58 ± 200.51 pA (BQ-788, Fig. 3*C*, red line) at −60 mV. Furthermore, ET-1 does not inhibit NPPB-sensitive Cl^−^ currents in the presence of BQ-788 (−1251.92 ± 250.83 pA, Fig. 3*C*, blue line), but it does inhibit Cl^−^ currents in the presence of BQ-123 (−817.52 ± 154.76 pA, Fig. 3*A*, blue line). Data graphs (Fig. 3, *B* and *D*, n = 5), with individual data points at −60 mV, confirm that ET-1 suppression of DCT1 NPPB-sensitive Cl^−^ currents is mediated by ET_B_ rather than ET_A_ receptors.

### ET-1 inhibits NPPB-sensitive Cl^−^ currents in DCT1 through nitric oxide (NO) pathway

**Figure 4:**
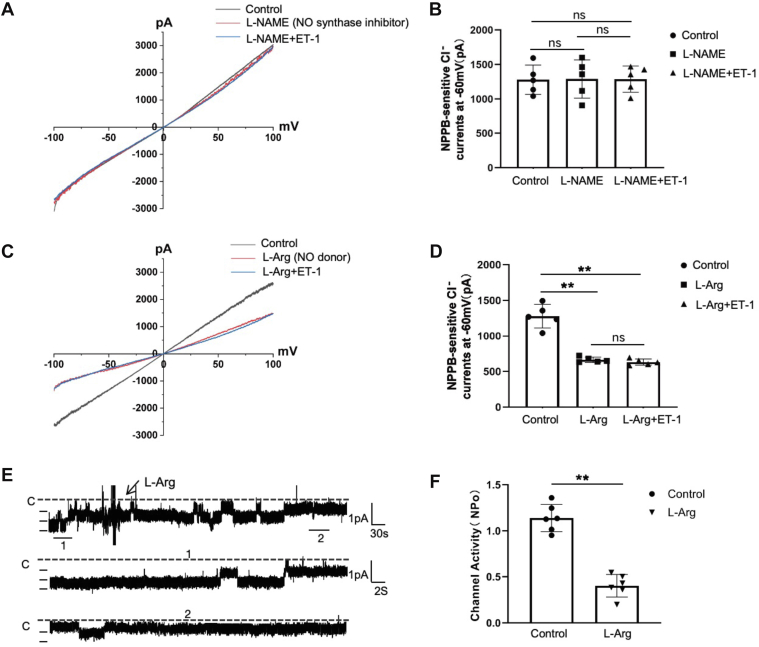
**ET-1 inhibits the NPPB-sensitive Cl^−^currents of DCT1 by stimulating NO.***A* and *C*, represent a whole-cell recording of NPPB-sensitive Cl^−^ currents on DCT1 under different conditions with ramp protocol. The *black line* shows the NPPB-sensitive Cl^−^ currents without any treatment. The *red line* indicates the effect of 100 μM L-NAME (*A*) or 1 mM L-Arg (*C*) on Cl^−^ currents. The blue line depicts the influence of 200 nM ET-1 on NPPB-sensitive Cl^−^ currents in DCT1 in the presence of L-NAME (*A*) or L-Arg (*C*). L-NAME, L-Arg, and ET-1 were introduced to the bath prior. Each experiment was conducted on different DCT1 cells. *B* and *D*, Bar graphs illustrate the data for each experiment, recorded *via* whole-cell technique at −60 mV. *E*, a single-channel recording shows the effect of 1 mM L-Arg on the 10 pS Cl^−^channels in DCT1. The experiments were conducted in cell-attached patches with a holding potential of −60 mV. The top trace displays the time course of the experiments. Three segments of the recording, marked by numbers, are expanded to exhibit the fast time resolution. The channel closed line is denoted by a dotted line with a "C". *F*, a scatter plot summarizes the single-channel results. The significance is evaluated using a one-way ANOVA method in whole-cell recording experiments, whereas a paired Student's *t* test is utilized in single-channel recording. All values are means ± SD; "ns" means no significance. ∗∗ indicates *p* < 0.01. L-Arg: L-arginine, NO donor; L-NAME: NO synthase inhibitor.

After showing that ET-1 inhibits NPPB-sensitive Cl^−^ currents *via* the activation of ET_B_ receptors, we explored the cellular signaling pathways affected by ET-1. The stimulation of ET_B_ receptors have been shown to trigger NO production (17, 18). Next, we investigated whether NO mediates ET-1 inhibition on Cl^−^ currents. We pre-incubated DCT1 in the bath with NO synthase (NOS) inhibitor L-NAME or NO donor L-arginine (L-Arg) and examined these effect on NPPB-sensitive Cl^−^ currents. Figure 4 *A/C* presents the currents curve in different isolated DCT1 by whole-cell recording with ramp protocol. When compared to the control (black line), the NPPB-sensitive Cl^−^ currents in DCT1 treated with L-NAME (100 μM) were not altered, the currents from −1280.02 ± 231.26 pA to −1290.90 ± 276.64 pA at −60 mV (Fig. 4*A*, red line). However, Figure 4*C* shows that L-Arg (1 mM) reduces NPPB-sensitive Cl^−^ currents from −1280.02 ± 165.92 pA (black line) to −664.14 ± 36.17 pA (red line). Meanwhile, we utilized single-channel recording to examine the effect of L-Arg on the 10-pS Cl^−^ channel. Figure 4*E* depicts that L-Arg (1 mM) inhibited the 10-pS Cl^−^ channel activity, the NPo from 1.14 ± 0.15 to 0.40 ± 0.12 (Fig. 4*F*, n = 5). We next examined the effect of ET-1 on ClC-Kb in the presence of L-NAME or L-Arg. The results are presented in Figure 4A/*C* with the blue line. The DCTs were pre-treated with L-NAME, L-Arg, and ET-1. As shown in Figure 4*A*, with L-NAME present, ET-1 failed to inhibit the NPPB-sensitive Cl^−^ currents, the currents are −1288.76 ± 190.29 pA. Although L-Arg inhibits ClC-Kb, application of ET-1 did not further decrease the NPPB-sensitive Cl^−^ currents, and there are −623.47 ± 17.82 pA at −60 mV (Fig. 4*C*). Data summarized in Figure 4, *B* and *D*, with individual data points at −60 mV (n = 5), confirm that ET-1 suppression of the DCT1 NPPB-sensitive Cl^−^ currents is mediated by the NO pathway.

### NO-related products diminished the ET-1-induced inhibition of NPPB-sensitive Cl^−^currents

**Figure 5:**
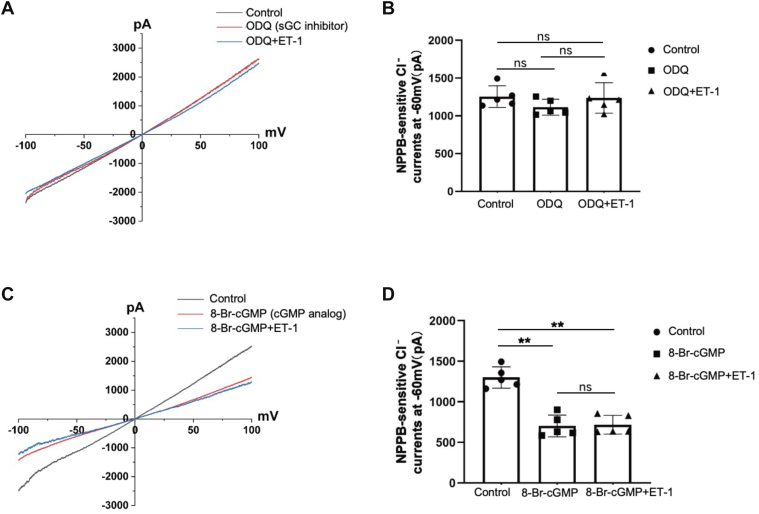
**The sGC-cAMP pathway mediates the effect of ET-1 on NPPB-sensitive Cl^−^currents in the DCT1**. *A*, whole-cell recording examines the effect of ET-1 on NPPB-sensitive Cl^−^ currents in DCT1 in the presence of 10 μM ODQ (sGC inhibitor, *blue line*) with ramp protocol. The *black line* denotes the typical NPPB-sensitive Cl^−^ currents. The *red line* illustrates the influence of ODQ alone on the Cl^−^ current. The drug was administered to the bath 5 to 7 min beforehand. *B*, Bar graphs illustrate the data for the NPPB-sensitive Cl^−^ current in each group for each experiment at −60 mV. *C*, Whole-cell recording shows the effect of ET-1 on NPPB-sensitive Cl^−^ currents in DCT1 in the presence of 1 mM 8-Br-cGMP (cGMP analog, *blue line*), using a ramp protocol. The *black line* represents the typical NPPB-sensitive Cl^−^ currents. The *red line* depicts the effect of 8-Br-cGMP on the Cl^−^ current. The drug was introduced into the bath 5 to 7 min prior. *D*, Bar graphs illustrate each experiment in each group at −60 mV. Experiments were conducted on different DCT1 cells. The significance is evaluated using a one-way ANOVA. All values are means ± SD; "ns" means no significance. ∗∗ indicates *p* < 0.01. sGC, soluble guanylate cyclase; cGMP, cyclic guanosine monophosphate.

It is known that NO interacts with soluble guanylate cyclase (sGC) to convert guanosine triphosphate (GTP) into cyclic guanosine monophosphate (cGMP) (19). We next studied the role of sGC and cGMP in mediating the effect of ET-1 on Cl^−^ currents in DCT1. Figure 5 illustrate the NPPB-sensitive Cl^−^ currents recorded under various conditions using whole-cell patch-clamp recordings with the ramp protocol. Figure 5*A* shows that, compared to the control (black line), the sGC inhibitor ODQ (10 μM) does not affect the Cl^−^ currents (red line). The NPPB-sensitive Cl^−^ currents from −1255.68 ± 1443.09 pA to −1215.60 ± 309.85 pA at −60 mV. Whereas the cGMP analog 8-Br-cGMP (1 mM) greatly decreased the Cl^−^ currents from −1300.02 ± 130.28 pA to −703.42 ± 134.04 pA (Fig. 5*C*, red line). Moreover, the treatment of DCT1 with ODQ or 8-Br-cGMP eliminated the effect of ET-1 on NPPB-sensitive Cl^−^ currents (ODQ + ET-1, -1237.06 ± 201.45 pA; 8-Br-cGMP + ET-1, -718.82 ± 170.73 pA; Fig. 5, A/*C*, blue line, n = 5). The experiments were performed on different separate DCT segments, with the drugs having been pre-added to the bath for 5 to 7 min. A summary of the data in Figure 5, *B* and *D* (n = 5), with each point represented at −60 mV, confirms that ET-1 suppresses DCT1 NPPB-sensitive Cl^−^ current through NO-related products.

### Phosphodiesterase 2 (PDE2) mediates the effect of ET-1 on NPPB-sensitive Cl^−^ currents

**Figure 6:**
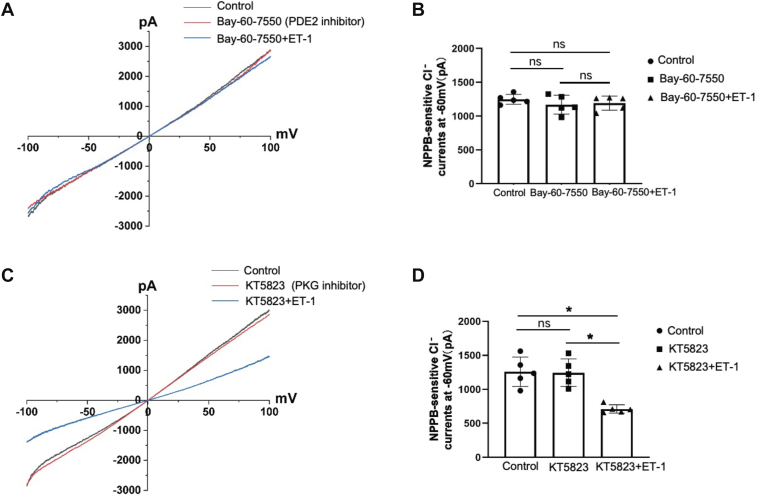
**ET-1 inhibits the NPPB-sensitive Cl^−^ currents in DCT1 by activating PDE2**. *A*, whole-cell recording examines the effect of 200 nM ET-1 on NPPB-sensitive Cl^−^ currents in DCT1 in the presence of 10 μM Bay-60-7550 (PDE2 inhibitor, *blue line*) with ramp protocol. The *black* currents is the normal NPPB-sensitive Cl^−^ currents. The *red line* shows the effect of Bay-60-7550 on NPPB-sensitive Cl^−^currents. *B*, graphs illustrate each experiment in which the effect of Bay-60-7550, Bay-60-7550+ET-1 on NPPB-sensitive Cl^−^ currents is examined at −60 mV. *C*, whole-cell recording examines the effect of 200 nM ET-1 on NPPB-sensitive Cl^−^ currents in DCT1 in the presence of 1 μM KT5823 (PKG inhibitor, *blue line*) with ramp protocol. The *black* currents represent the normal NPPB-sensitive Cl^−^ currents. The red line indicates the effect of KT5823 on NPPB-sensitive Cl^−^ currents. *D*, graphs illustrate each experiment in which the effect of KT5823, KT5823+ET-1 on NPPB-sensitive Cl^−^ currents is examined at −60 mV. Each experiment was conducted on different DCT1 cells. The significance is evaluated using a one-way ANOVA. All values are means ± SD; "ns" means no significance. ∗ indicates *p* < 0.05. PKG, Protein kinase G; PDE2, Phosphodiesterase 2.

Protein kinase G (PKG) and PDE2 can be activated by cGMP (20, 21). We then examine whether the PKG or PDE2 pathway is engaged in regulating the effect of ET-1 on Cl^−^ currents in DCT1. Figure 6*A*/C is the recordings showing the NPPB-sensitive Cl^−^ currents measured with ramp protocol before and after ET-1 treated with PKG inhibitor KT-5823 (1 μM) or the PDE2 inhibitor Bay-60-7550 (10 μM). We conducted experiments on different DCT1 cells, with KT-5823, Bay-60-7550, or ET-1 added to the bath in advance. As compared to the control (Fig. 6A/*C*, black line), the NPPB-sensitive Cl^−^ currents treated with Bay-60-7550 or KT-5823 were not altered; they are −1169.74 ± 138.61 pA (Fig. 6*A*, red line) and −1244.38 ± 202.67 pA (Fig. 6*C*, red line) at −60 mV. However, in the presence of (Bay-60-7550), ET-1 was unable to inhibit the NPPB-sensitive Cl^−^ currents (−1191.90 ± 104.34 pA, Fig. 6*A*, blue line). With KT-5823 present, ET-1 still inhibited the NPPB-sensitive Cl^−^ currents (−711.26 ± 61.08 pA; Fig. 6*C*, blue line). An overview of the data depicted in Figure 6, *B* and *D* (n = 5), where each point is indicated at −60 mV. These results suggest that ET-1 inhibits the NPPB-sensitive Cl^−^ currents *via* the PDE2 rather than the PKG pathway.

### ET-1 inhibits NCC expression and phosphorylation

**Figure 7:**
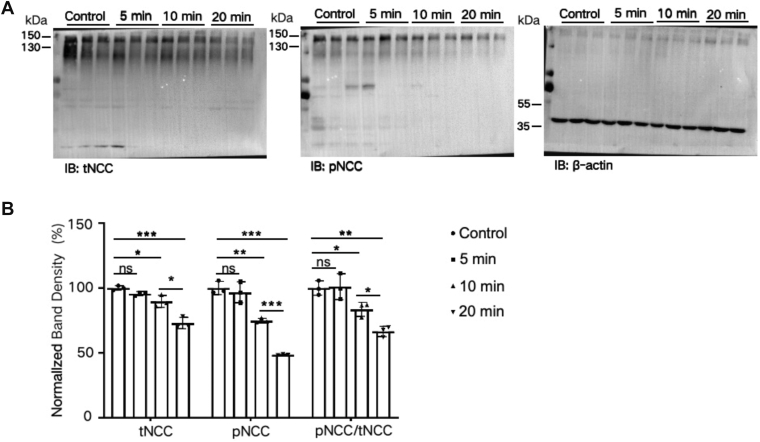
**ET-1 inhibits the expression of tNCC and pNCC**. *A*, a set of Western blots displays the expression of tNCC and pNCC (at Thr^53^) in kidney cortical tissue treated with ET-1 (200 nM) for 5, 10, and 20 min. *B*, bar graphs summarize the normalized band intensities of tNCC and pNCC, respectively (n = 3 male mice). All values are means ± SD; "ns" means no significance. *∗p* < 0.05*,∗∗p <* 0.01*,∗∗∗p* < 0.001 by one-way ANOVA. And the Holm-Sidak test was employed as a *post hoc* analysis.

The Cl^−^ channel activity in DCT1 influences [Cl^−^]_i_, inversely related to WNK4, which regulates NCC activity (1). We hypothesized that ET-1 would inhibit NCC expression, if the NPPB-sensitive Cl^−^ currents are important for WNK4-mediated modulation of NCC. To test this hypothesis, we examined the effect of ET-1 on the expression of and total NCC (tNCC) and phosphorylated NCC (pNCC at Thr^53^). Figure 7*A* depicts a Western blot showing the effect of ET-1 (200 nM) on the expression of tNCC and pNCC. It shows that ET-1 treatment for 5 min does not affect tNCC and pNCC levels but significantly inhibits tNCC and pNCC after 10 min (90% ± 5% for tNCC; 75% ± 2% for pNCC; Fig. 7*B*) and becomes more marked after 20 min (73% ± 4% for tNCC; 49% ± 0.4% for pNCC; Fig. 7*B*). Thus, the data indicate that ET-1 induces inhibition of tNCC and pNCC expression.

**Figure 8:**
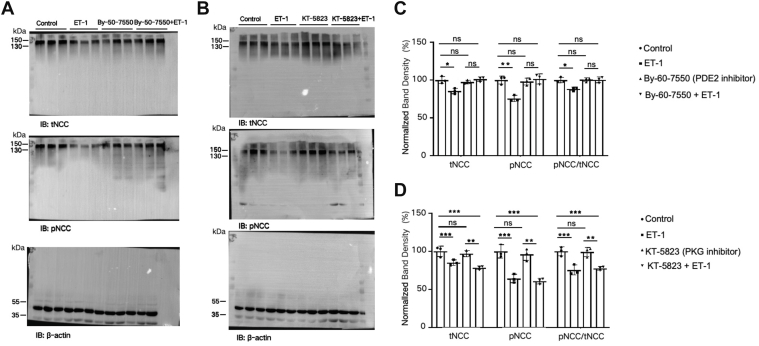
**ET-1 decreases protein expression of tNCC and pNCC *via* the Phosphodiesterase 2 (PDE2) pathway**. Kidney cortical tissue from 3 male mice were treated with ET-1 (200 nM) for 10 min. *A*, protein expression bands for the action of ET-1 on tNCC and pNCC in the presence of 10 μM Bay-60-7550 (PDE2 inhibitor). *B*, protein expression bands for the action of ET-1 on tNCC and pNCC in the presence of 1 μM KT-5823 (PKG inhibitor). *C*, quantification of the bands obtained from the western blots in (*A*). *D*, quantification of the bands in the western blots from (*B*). All values in Bar graphs are means ± SD; "ns" means no significance. *∗p <* 0.05*,∗∗p <* 0.01*,∗∗∗p <* 0.001 by two-way ANOVA. n = 3 male mice. PKG, Protein kinase G; PDE2, Phosphodiesterase 2.

Using whole-cell patch clamping, we demonstrated that PDE2 mediates ET-1 regulation of NPPB-sensitive Cl^−^ channels in DCT1. To investigate whether the regulation of NCC expression by ET-1 involves the PDE2 pathway, we then used immunoblotting to examine the effect of ET-1 on the expression of tNCC and pNCC in the presence of PDE2 or PKG2 inhibitors. Figure 8*A* shows a Western blot revealing that with the administration of the PDE2 inhibitor Bay-60-7550 (10 μM), ET-1 fails to alter the expression levels of tNCC and pNCC. In the presence of the PKG inhibitor KT-5823 (1 μM), the ET-1-mediated decline in the expression levels of tNCC (78% ± 2% of control) and pNCC (61% ± 4% of control) is sustained (Fig. 8*B*). Figure 8, *C* and *D* are bar graphs summarizing the normalized band density of Western blots. These findings imply that the inhibitory effect of ET-1 on NCC expression and phosphorylation is mediated through the PDE2 pathway.

## Discussion

Endothelin-1 (ET-1) contributes to hypertension by vasoconstriction, but it also lowers blood pressure through its diuretic and natriuretic effects (22). The hypertensive effect of ET-1 involves ET_A_ receptors, while its hypotensive effect is mediated by ET_B_ receptors, which are located in the vascular endothelial cell membrane and renal tubular epithelial cell membrane. Activation of ET_B_ receptors by ET-1 results in vasodilation and reduced NaCl reabsorption in renal tubules, contributing to blood pressure reduction (23). The mechanism by which ET-1 inhibits NaCl reabsorption in renal tubules has been thoroughly investigated. For example, ET-1 activates the ET_B_ receptors, decreases the activity of key transporters involved in NaCl handling, including sodium-hydrogen exchanger isoenzyme 3 (NHE3) and Na-K-ATPase in the proximal tubule (PT), Na-K-2Cl cotransporter (NKCC2) in the TAL, and ENaC in the CD (2, 24, 25). However, it is unclear whether ET-1 regulates NaCl reabsorption associated with DCT (22). Wendel *et al.* did not observe ET_B_ receptor expression in the DCT of the rat kidney (14). However, our study confirms ET_B_ receptors exist in the DCT1 of mice. Tokonami *et al.* detected ET_B_ mRNA in the DCT of mice when studying the role of ET-1 in vitamin D-induced hypercalcemia (13), which partially supports our findings. In addition, we demonstrate that ET-1 inhibits the ClC-K2 channel currents in DCT1, and the effect is blocked by the ET_B_ receptor blocker. Our data suggest a potential new mechanism by which ET-1 inhibits NaCl reabsorption in renal tubules.

In renal tubular cells, several studies have confirmed that ET-1 acts by activating ET_B_ to produce NO (17). Plato *et al.* demonstrated that ET-1 inhibits NaCl reabsorption in the cortical TAL *via* the NO pathway mediated by ET_B_ (8). Herrera *et al.* reported that ET_B_ activates the phosphatidylinositol 3-kinase (PI3K), Protein kinase B (PKB/Akt), and endothelial nitric oxide synthase (eNOS) pathways, leading to an increase in NO production in the medullary TAL, and then inhibiting NKCC2 activity (26). Stricklett *et al.* found that ET-1 stimulates NO production in the inner medullary CD of rats, reducing ENaC activity (25). Wang *et al.* discovered that low concentrations of L-NAME suppress NO production, leading to inappropriate activation of NCC in DCT and salt-sensitive hypertension (27). According to our study, we confirm that the inhibition of NPPB-sensitive Cl^−^channel currents by ET-1 in DCT1 is related to the NO-sGC-cGMP signaling pathway. We demonstrate that: (1) the NOS inhibitor L-NAME effectively blocks the ET-1 induced inhibition of NPPB-sensitive Cl^−^ currents; (2) the NO activator L-Arg directly inhibits the ClC-K2, with no additional suppression by ET-1; (3) sGC inhibitor eliminates ET-1 mediated inhibition; and (4) cGMP analogs reduce NPPB-sensitive Cl^−^currents, preventing ET-1-induced inhibition. As a result of these experiments, ET-1 inhibits the NPPB-sensitive Cl^−^ currents by activating the NO-sGC-cGMP pathway.

The activation of cGMP induces the production of PDE2 and PKG (20, 21). Wu *et al.* demonstrated that NO inhibits 10 pS Cl^−^ channels activity on the basolateral of the TAL *via* the cGMP/PKG pathway (28). In addition, they identified that the cGMP/PKG pathway also mediates the inhibition of Cl^−^channels by S-Nitroso-N-acetyl penicillamine (SNAP) (29). In order to determine whether ET-1 inhibits the NPPB-sensitive Cl^−^ currents in DCT1 *via* the cGMP/PKG pathway, we employed the PKG inhibitor KT5823 in whole-cell patch clamp experiments. The findings showed that KT5823 did not prevent inhibition of ET-1. However, PDE2 inhibitor eliminated the inhibitory effect of ET-1. That indicates the regulation of the NPPB-sensitive Cl^−^ currents by ET-1 is mediated by cGMP-PDE2 rather than the cGMP-PKG pathway. The TAL and DCT Cl^−^ channels are members of the ClC family (1), but the mechanisms of NO-activated cGMP regulation of Cl^−^ channels are different. The reason for this is that in addition to the different properties of the cells, the current recording method and drug concentration may also be important factors. Our experiment utilized whole-cell patch clamp recording. The concentrations of ET-1 were high, potentially leading to increased production of NO and cGMP. It has been demonstrated that cGMP decreases cyclic adenosine monophosphate (cAMP) levels *via* the PDE2 (20), and the ability to hydrolyze cAMP activity of PDE2 increases approximately 5-fold in the presence of cGMP (30). cAMP regulates several ion channels, including specific chloride channels such as CFTR and ClC-K1 (31, 32). Shintani *et al.* observed that cAMP activates NPPB-sensitive Cl^−^ currents in renal epithelial A6 cells through both protein kinase A (PKA)-dependent and independent pathways (33). Alés *et al.* provided evidence that cGMP influences NKCC2 in the TAL and decreases chloride flux *via* the cGMP-PDE2 pathway (34). Furthermore, cAMP promotes NaCl reabsorption in the DCT (35). Therefore, PDE2 degradation of cAMP, leading to reduced cAMP levels, may explain the inhibition of the NPPB-sensitive Cl^−^ currents by ET-1 in the DCT. However, some studies suggest that cAMP does not directly regulate Cl^−^ channels (2). Loudel *et al.* study reveals that Cl^−^ channel in the basolateral membrane of DCT is not regulated by cAMP/PKA (2). It looks like cAMP and PKA do not regulate Cl^−^ channel activity directly in DCT cells. Nevertheless, it does not exclude the possibility of reduced intracellular cAMP levels decreased Kir4.1/5.1 channels or CFTR channels of the DCT. That could influence the Cl^−^ channel activity by lowering [Cl^−^]_i_. Moreover, PDE2 may inhibit Cl^−^ channel currents in other ways.

Except for the proximal tubule, NaCl reabsorption in the kidney is mediated by basolateral chloride channels (1). Chloride channels in the renal tubule belong to the ClC family, which includes CLC-Ka and CLC-Kb, also known as ClC-K1 and ClC-K2 in rodents (1). The DCT reabsorbs a certain amount of NaCl from the filtrate. In mice kidneys, it is divided into DCT1 and DCT2 (36, 37). In DCT1, NaCl is reabsorbed in two steps. First, Na^+^ and Cl^−^ enter the cell through the NCC, which is located on the apical membrane and sensitive to thiazide diuretics. Secondly, Na^+^ is transported to the interstitial fluid by the Na-K-ATPase, and Cl^−^ is reabsorbed into the interstitial fluid through chloride channels present on the basolateral membrane (1, 2, 38). Immunofluorescence and patch-clamp techniques have identified ClC-K2 on the basolateral membrane of DCT1, with a conductance of 10-pS (39). Kidney-specific *Clcnk2*^*−/−*^ knockout of DCT, there were no other Cl^−^ channel currents observed on DCT, and thiazide diuretics did not work (5). Su *et al.* demonstrated that the inhibition of Cl^−^ current using NPPB led to a significant increase in the [Cl^−^]_i_ in DCT cells, and subsequently inhibited the WNK4 system, resulting in a reduction in NCC activity (40). Our previous study showed that the absence of Kir4.1/5.1 channels in the basolateral membrane of DCT1 resulted in cell membrane depolarization, which decreased the driving force for Cl^−^ transport, reduced Cl^−^ channel current, inhibited NCC activity, and increased urinary sodium excretion (41). Thus, we showed that ET-1 inhibits the NPPB-sensitive Cl^−^ current of DCT in this study, it might increase [Cl^−^]_i_ and inhibit the NCC. We also investigated the regulation of ET-1 on tNCC, pNCC expression. The results indicated that these protein levels were not altered by exposure to ET-1 in DCT tissues for 5 min. After 10 min, their expression was significantly inhibited. This is different from the rapid changes in ClC-K2 currents in patch clamp experiments. It is possible that in those electrophysiology experiments, ET-1 was directly applied to Cl^−^ currents in a single DCT tubule.

**Figure 9:**
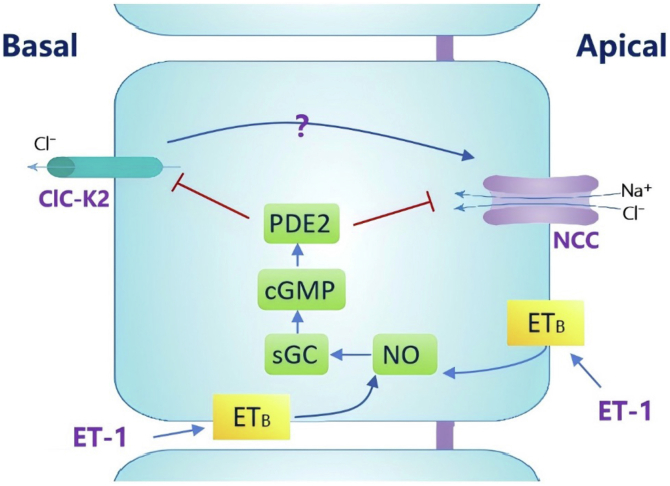
**The mechanism of ET-1 inhibit Cl^−^ channel and NCC in DCT**. ET-1 inhibits NCC and ClC-K2 in DCT by NO-sGMP-phosphadiesterase-2 dependent-pathway. ClC-K2, the NPPB-sensitive Cl-channel, 10-pS Cl^−^ channel; NO, Nitric Oxide; sGC, soluble guanylate cyclase; cGMP, cyclic guanosine monophosphate; PDE2, phosphodiesterase 2.

Our results show that ET-1 inhibits the NPPB-sensitive Cl^−^ currents and NCC in DCT1 through activation of NO-cGMP-PDE2 pathway (Fig. 9). This may be a novel mechanism for ET-1 to increase urinary sodium excretion. Additionally, a high-salt diet can enhance ET-1 production (42). We confirmed that ET-1 inhibits NCC, indicating that the stimulating effect of a high-salt diet on urinary sodium excretion may involve NCC regulation by ET-1.

## Experimental procedures

### Animals

This experiment was conducted using 4 to 6-week-old male C57BL/6J mice, which were provided by Zhuhai BestTest Biotechnology Co., Ltd under license No. SCXK(GD)2020-0051. During the experimental period, the mice were maintained on a normal diet with free access to water. The study received approval from the Experimental Animal Welfare Ethics Committee of Zunyi Medical University (No. ZMU21–2303–190).

### Preparation of DCTs

The mice were sacrificed *via* cervical dislocation. Upon opening the abdomens of the mice, 2 ml of Leibovitz's L-15 medium (Wuhan Pricella Biotechnology Co. Ltd) containing collagenase II (1 mg/ml, Worthington Biochemical Corporation) was perfused into the left kidney. After perfusion, the left kidney was excised, and the renal cortex was preserved. We then sliced the kidney into small pieces (1 mm) and incubated them in collagenase-containing L-15 medium for 30 to 50 min at 37 °C. After incubation, the kidney tissue was washed three times in fresh Leibovitz's L-15 medium. Utilizing the Nikon SM2745 microscope (Nikon), we separated the DCT1. The DCT1 is located near the glomerulus (refer to Fig. S1). We placed the isolated DCT1 tubules on a small cover glass coated with polylysine (Sigma-Aldrich) and transferred the cover glass to an inverted microscope (Nikon, Eclipse Ti2-U) equipped with a chamber suitable for patch-clamp experiments.

### Patch-clamp experiment

For whole-cell recording, we used a Narishige electrode puller to pull pipettes from borosilicate glass (BF 150–110–10, Sutter), which had a resistance of 2 MΩ when filled with pipette solution. An Axon 700B patch-clamp amplifier was utilized for recording. Currents were low-pass filtered at 1 kHz and digitized by an Axon interface with a 4 kHz sampling rate (Digidata 1550B). The pipette solution contained (in mM) 140 KCl, 2 MgCl_2_, 1 EGTA, and 5 HEPES (pH 7.4), while the bath solution comprised (in mM) 140 NaCl, 5 KCl, 1.8 MgCl_2_, 1.8 CaCl_2_, and 10 HEPES (pH 7.4). The whole-cell Cl^−^ current was determined by adding 10 μM NPPB (MCE, USA) to the bath solution. Data were analyzed using the pCLAMP Software System 11.2 (Axon). The Fig. S2 illustrates a typical example of whole-cell recordings before and after the application of 10 μM NPPB, using a ramp protocol from −100 to 100 mV. The NPPB-sensitive Cl^−^currents were obtained by subtracting the whole-cell currents recorded after NPPB addition from those recorded before addition. When examining the impact of ET-1 or other reagents on DCTs, we initially introduce the medication into the bath for a duration of 5 to 7 min, followed by the recording of NPPB-sensitive Cl^−^ currents using identical methods.

For single-channel recording, pipettes (BF 150–86–10, Sutter) were utilized and the resistance is 5 MΩ when filled with the following pipette solution. For Cl^−^ singel channel, the pipette solution was composed of 140 mM NaCl, 1.8 mM MgCl_2_, and 10 mM HEPES, whereas the bath solution comprised (in mM) 140 NaCl, 5 KCl, 1.8 MgCl_2_, 1.8 CaCl_2_, and 10 HEPES. All solutions were adjusted to pH 7.4 using NaOH. The Axon 200B patch-clamp amplifier was employed for Cl^−^single-channel recordings. Currents were low-pass-filtered at 1 kHz and digitized by an Axon interface (Digidata 1550B) at a sampling rate of 4 kHz. Cl^−^ channel activity defined as NPo (the product of channel numbers and channel-open probability), was derived from 60s data samples in the steady state according to the formula:

$$\text{NPo}=\Sigma \;\left(t1+2t2+\ldots {\text{it}}_{i}\right)$$

where *t*_*i*_ denotes the fractional open time spent at each of the observed current levels. Data analysis was performed using the pCLAMP Software System 11.2 (Axon). Fig. S3 provides a representative example of the calculation process for determining Cl^−^ channel NPo.

### Immunofluorescence

Mice were anesthetized with 2% halothane, and their abdomens were opened to facilitate rapid perfusion with saline. Subsequently, the mice were perfused with 40 ml of 4% paraformaldehyde (Beyotime Biotechnology). After that, the kidneys were excised and post-fixed in 4% paraformaldehyde for 24 h. The kidneys were then dehydrated and embedded in paraffin. The tissues were cut into 3 μM slices using a fully automated rotary microtome (Leica RM2255). Slices were mounted onto glass slides and baked at 65 °C for 90 min. Following this, they underwent dewaxing and rehydration, followed by antigen retrieval. The tissue was incubated with a blocking buffer containing 3% BSA for 1 h and then rinsed with 1× PBS for 15 min. It was then incubated with primary antibodies (rabbit anti-ET_B_, 1:200, Abcam; guinea pig anti-Parvalbumin, 1:100, Swant) at 4 °C for 18 h. The slides were thoroughly washed with 1×PBS and then followed by the addition of a mixture of secondary antibodies in 3% BSA for 2 h at room temperature. The information on the antibodies used for immunofluorescence staining is provided in Table S1.

### Western blot

The total proteins from the renal cortex were homogenized in ice-cold solutions supplemented with phosphatase and protease inhibitor cocktails (Bimake). Subsequently, the protein concentration was measured using the BCA protein assay kit (Shanghai Beyotime Biotechnology). The proteins were then separated on an 8% Tris-glycine gel and transferred to a nitrocellulose membrane. The membrane was blocked with 5% BSA for 1 h, rinsed with TBS-T for 10 min, and incubated overnight at 4°C with the respective primary antibodies (β-actin 1:5000, Cell Signaling Technology; NCC 1:2000, Millipore; pNCC at Thr^53^ 1:1000, Abcam). The Odyssey infrared imaging system (LI-COR) was used to scan the membrane, and imaging was conducted using the chemiluminescence imaging system provided by QuickChemi 5200 (Monad Biotechnology). The information on antibodies used for immunoblotting is in Table S1.

### Experimental materials and statistics

ET-1, L-Arginine, ODQ, 8-Br-cGMP, KT-5823, and Bay-60-7550 were obtained from MCE, while BQ-123 and BQ-788 were purchased from Sigma. L-NAME was sourced from Glpbio. The statistical analysis was conducted using SPSS version 29.0 (IBM Corp.). A *t* test was utilized to analyze the values between two groups, and a paired *t* test was employed for comparisons of values within the same group. One-way or two-way ANOVA was used to analyze the results of more than two groups, and the Holm-Sidak test was employed as a *post hoc* analysis. We considered the difference to be statistically significant if the *p*-value was less than 0.05. Data are presented as the mean ± SD.

## Data availability

The data that support the findings of this study are available on request from the corresponding author.

## Supporting information

This article contains supporting information.

## Conflict of interest

The authors declare that they have no conflicts of interest with the contents of this article.
