# Supplementary material for: Nitric oxide mediates ET-1-induced-inhibition of NPPB-sensitive Cl− currents in the early distal convoluted tubule of the mouse kidney
Source: J Biol Chem. 2026 Jan 23;302(3):111202. doi: 10.1016/j.jbc.2026.111202 (PMC12925561; doi:10.1016/j.jbc.2026.111202)
Supplement: Supporting information [file mmc1.pdf]

Supplemental Material

Nitric Oxide Mediates ET-1-induced-Inhibition of NPPB-Sensitive Cl^- Currents in Early Distal Convoluted Tubule of the Mouse Kidney

- Supplemental Figure 1. Image of isolated distal nephrons（DCT）for the patch-clamp experiments
- Supplemental Figure 2. NPPB-sensitive Cl^- currents in DCT1
- Supplemental Figure 3. A sample for calculating 10 pS Cl^- channel NPo
- Supplemental Figure 4. Single-channel recording demonstrating the effect of ET-1 on the 10-pS Cl channels in the male and female DCT1
- Supplemental Table 1. Information of Antibodies used for immunoblotting and fluorescence staining

Supplemental Figure 1. Image of isolated distal nephrons (DCT) for the patch-clamp experiments.

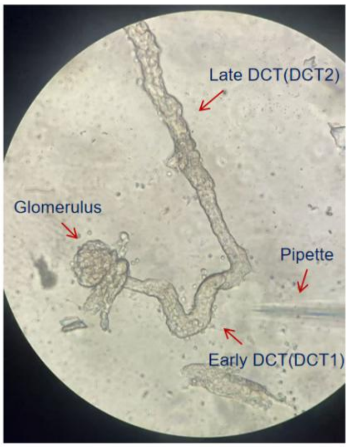

Supplemental Figure 2. NPPB-sensitive Cl$^-$ currents in DCT1. A whole-cell recording shows the currents before adding 10μM NPPB, after adding NPPB, the net NPPB-sensitive Cl$^-$ currents in DCT 1 measured with ramp protocol from -100 to 100 mV.

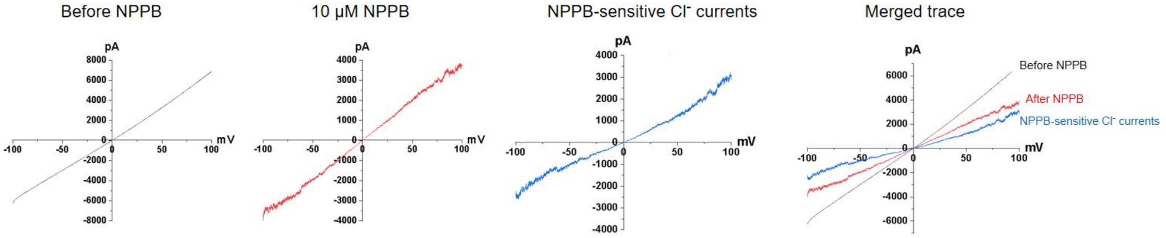

Supplemental Figure 3. A sample for calculating 10 pS Cl$^-$ channel NPo.Analysis of 10 pS Cl$^-$ channel NPo using pCLAMP Software System 11.2. The channel recording of the 10 pS Cl$^-$ channel in low panel is from a part of trace presented. Statistical information summarizes the results including NPo (marked by a red line) and channel amplitude.

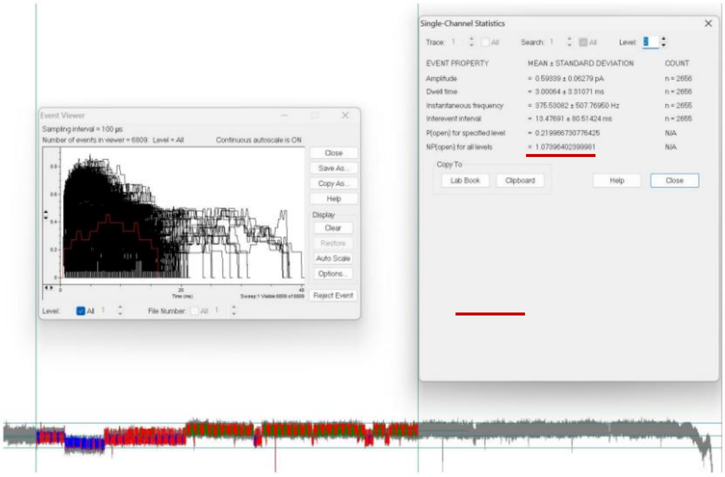

Supplemental Figure 4

Single-channel recording demonstrating the effect of 200 nm ET-1 on the 10-pS Cl channels in male and female DCT1. Figure A is male mice and Figure B is female mice. The experiments were conducted in a cell-attached patch with a holding potential of −60 mV. The top trace illustrates the experimental timeline, with two segments of the trace, marked by numbers, expanded to exhibit the fast temporal resolution. The channel closed state is denoted by a dotted line and the letter 'C'. (C) The bar graph summarizes the effect of ET-1 on the 10 pS Cl^- channel in male or female mice. All values are means ± SD. An asterisk indicates a significant difference compared with the control value (*:P<0.05,**: P < 0.01).

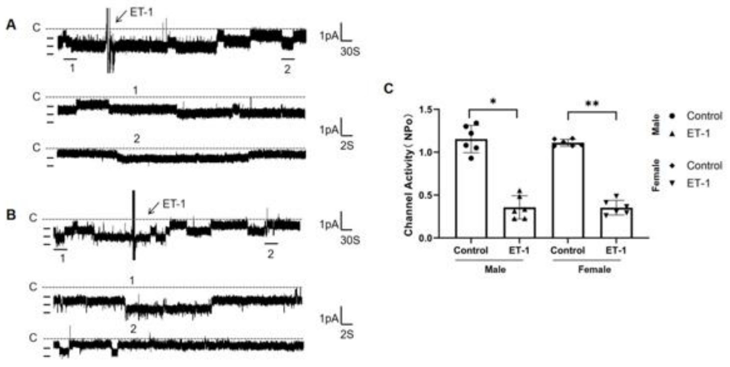

Supplemental Table 1. Information of Antibodies used for immunoblotting and fluorescence staining

Antibody	Species	Dilution for WB	Dilution for IF	Source
ET_B (ab117529)	Rabbit		1：100	Abcam
Alexa Fluor 555（A0453)	Donkey		1：400	Beyotime
Parvalbumin（PV27)	Guinea pig		1：100	Swant
Alexa Fluor 488 (ab175678)	Goat		1：400	Abcam
β-actin（4970S)	Rabbit	1：5000		Cell Signaling Technology
NCC（AB3553)	Rabbit	1：2000		Millipore
pNCC（ab254039)	Rabbit	1：1000		Abcam
